# Supplemental Effects of Functional Oils on the Modulation of Mucosa-Associated Microbiota, Intestinal Health, and Growth Performance of Nursery Pigs

**DOI:** 10.3390/ani11061591

**Published:** 2021-05-28

**Authors:** Vitor Hugo C. Moita, Marcos Elias Duarte, Suelen Nunes da Silva, Sung Woo Kim

**Affiliations:** Department of Animal Science, North Carolina State University, Raleigh, NC 27695, USA; vccardos@ncsu.edu (V.H.C.M.); mduarte@ncsu.edu (M.E.D.); snunesd@ncsu.edu (S.N.d.S.)

**Keywords:** cashew nutshell liquid, castor oil, intestinal health, microbiota, nursery pigs, phytobiotics

## Abstract

**Simple Summary:**

The earlier establishment of a health-benefiting intestinal microbiota can be an important strategy to improve intestinal health and subsequent growth performance. Functional oils, such as castor oil and cashew nutshell liquid, have been studied for promoting intestinal health due to their antimicrobial and anti-inflammatory properties. This study aimed to investigate the benefits of supplementation of functional oils on modulation of mucosa-associated microbiota, enhancing the intestinal health and growth performance of nursery pigs. It was demonstrated that the functional oils enhanced the intestinal health of the pigs by increasing beneficial and reducing harmful bacteria and by potentially reducing jejunal oxidative stress and enhancing intestinal morphology. Our results suggest that the blend composed of castor oil and cashew nutshell liquid can be used in nursery pigs to modulate the jejunal mucosa-associated microbiota and intestinal integrity of nursery pigs.

**Abstract:**

This study aimed to investigate the effects of functional oils on modulation of mucosa-associated microbiota, intestinal health, and growth performance of nursery pigs. Forty newly weaned pigs (20 barrows and 20 gilts) with 7.0 ± 0.5 kg body weight (BW) were housed individually and randomly allotted in a randomized complete block design with sex and initial BW as blocks. The dietary treatments were a basal diet with increasing levels (0.00, 0.50, 0.75, 1.00, and 1.50 g/kg feed) of functional oils (a blend of castor oil and cashew nutshell liquid; Oligo Basics USA LLC, Cary, NC) fed to pigs for 34 days divided in two phases (P1 for 13 days and P2 for 21 days). Growth performance was analyzed weekly. On day 34, all pigs were euthanized to collect jejunal mucosa for analyzing the mucosa-associated microbiota and intestinal health, and ileal digesta for analyzing apparent ileal digestibility. Data were analyzed using SAS 9.4. Supplementation of functional oils did not affect the overall growth performance. Increasing supplementation of functional oils reduced (*p* < 0.05) the relative abundance of Helicobacteraceae, whereas it increased (*p* < 0.05) *Lactobacillus kitasatonis*. Supplementation of functional oils tended (*p* = 0.064) to decrease protein carbonyl and increase the villus height (*p* = 0.098) and crypt depth (*p* = 0.070). In conclusion, supplementation of functional oils enhanced intestinal health of nursery pigs by increasing beneficial and reducing harmful bacteria, potentially reducing oxidative stress and enhancing intestinal morphology, without affecting overall growth performance of pigs. Supplementation of functional oils at 0.75–1.50 g/kg feed was the most beneficial to the jejunal mucosa-associated microbiota and intestinal integrity of nursery pigs.

## 1. Introduction

Nursery pig’s face changes in dietary sources and the environment after weaning that leads to weaning stress increase the risk of post-weaning diarrhea (PWD) during the first week [1,2,3]. Post-weaning diarrhea is one of the most destructive problems to newly weaned pigs, impairing their intestinal health and subsequent performance [4,5]. Antimicrobial growth promoters (AGP) have been conventionally used in nursery diets to control PWD and improve growth performance [6]. However, with the increasing concern about antimicrobial-resistant pathogens [7] and the ban of using AGP in many countries [8], different alternatives have started to be developed to promote the health and growth of pigs [9,10].

Phytobiotics including essential oils and herbal extracts have been studied as alternatives to AGP for swine and poultry [11,12,13,14] to promote functions beyond their energy value such as restoring the impaired villus-crypt structure as well as improving carcass characteristics [15,16,17]. Castor oil, which is mainly composed of ricinoleic acid, is known to have antimicrobial and anti-inflammatory effects when ingested orally by guinea pigs [18]. However, castor oil at oral doses greater than 10 mL/d can cause laxative effects, limiting its application in animal feeds at high doses [19,20,21]. Cashew nutshell liquid contains alkylphenolic oil, mainly composed of anacardic acid, cardanol, cardol, and methylcardol [22], providing anti-inflammatory and antioxidative effects that may lead to a reduction of diarrhea [23]. A combination of castor oil and cashew nutshell liquid as a blend provided antimicrobial and antioxidant effects, enhanced growth performance and intestinal structure in poultry [16,17,19,20], and may also apply to nursery pigs susceptible to intestinal challenges upon weaning.

It has been hypothesized that dietary supplementation of functional oils can modulate the mucosa-associated microbiota, thus enhancing the intestinal health and growth performance of nursery pigs. The objective of this study was to investigate the supplemental effects of functional oils on the modulation of mucosa-associated microbiota, intestinal health, and growth performance of nursery pigs.

## 2. Materials and Methods

### 2.1. Animals, Design, and Diets

The experimental protocol was approved by the Institutional Animal Care and Use Committee of North Carolina State University. Forty newly weaned pigs at 25 days of age (20 barrows and 20 gilts) with an initial BW of 7.0 ± 0.5 kg were housed individually and randomly allotted to five dietary treatments based on a randomized complete block design with initial BW and sex as blocks. Five dietary treatments (*n* = 8) were increasing supplementation (0.00, 0.50, 0.75, 1.00, and 1.50 g/kg feed) of functional oils (a blend of castor oil and cashew nutshell liquid; Oligo Basics USA LLC, Cary, NC). A basal diet was formulated to meet the nutrient requirements of pigs suggested by NRC [24] (Table 1). Supplementation of functional oils replaced the same amount of corn in basal diets. Pigs had ad libitum access to feed and water. The experimental period was 34 d, which was divided into two phases: phase 1 (day 1 to 13) and phase 2 (day 13 to 34). Titanium dioxide (0.4%) was added to phase 2 diets as an indigestible external marker.

### 2.2. Growth Performance and Fecal Score

The BW of each pig and individual feed intakes were recorded at the end of each phase to calculate the average BW, ADG, ADFI, and G:F as indicators of growth performance. Fecal scores were recorded daily based on a 1 to 5 scale, as described by Duarte et al. [25]: (1) very firm stool, (2) normal firm stool, (3) moderately loose stool, (4) loose, watery stool, and (5) very watery stool.

### 2.3. Sample Collection

After 34 days of feeding, all pigs were euthanized to remove the gastrointestinal tract to collect jejunal mucosa to measure microbiota composition, immune status, and oxidative stress status; jejunal tissues to measure morphology and crypt cell proliferation; and ileal digesta to measure apparent ileal digestibility (AID) of nutrients. Mucosal samples from mid-jejunum (3 m after the pyloric duodenal junction) were scraped, placed into 2 mL tubes, and later stored at −80 °C (after snap-freezing in liquid nitrogen, immediately after collection) for the microbiome, immune, and oxidative stress analysis. Sections (5 cm) of the mid-jejunum were taken, flushed with a 0.9% saline solution, and placed into 50 mL tubes with 40 mL of 10% formalin to be fixed for further microscopic assessment of jejunal morphology. For measuring AID of dry matter (DM), crude protein (CP), and gross energy (GE), an ileal portion (a portion of 30 cm prior to the ileocecal valve) was used to obtain ileal digesta. The ileal digesta was collected into 150 mL containers, placed on ice, and then stored at −20 °C for further analysis. The sample collection procedures were performed as previously described by Duarte et al. [2].

### 2.4. Microbiome Sequencing

The DNA was extracted from jejunal mucosa samples for microbiome analysis, as previously described by Adhikari et al. [26]. The kit QIAamp Fast DNA Stool (Qiagen; Germantown, Maryland, USA) was used to perform the DNA extraction. Samples of extracted DNA were sent to Mako Medical Laboratories (Raleigh, NC, USA) for microbial sequencing using the 16S rRNA amplicon. Briefly, the samples were prepared for the template using the Ion Chef instrument, and sequencing was performed on the Ion S5 system (Thermo Fisher Scientific, Inc., Wilmington, DE, USA). The Ion 16S Metagenomics Kit (Thermo Fisher Scientific) was used to amplify variable regions V2, V3, V4, V6, V7, V8, and V9 of the 16S rRNA gene. To produce raw unaligned sequence data files, sequences were processed using the Torrent Suite Software (version 5.2.2) (Thermo Fisher Scientific). The Ion Reporter Software Suite (version 5.2.2) of bioinformatics analysis tools (Thermo Fisher Scientific) was used to perform the sequence data analysis in alignment with GreenGenes and MicroSeq databases, alpha and beta diversity plot generation, and the operational taxonomic unit (OTU) table generation. The Ion Reporter’s Metagenomics 16S workflow powered by Qiime (version w1.1) was used to analyze the samples. The relative abundance for phylum, family, species, and genus was calculated based on the OTU data. The “Others” were considered representing the combined OTU with a relative abundance of <1%, as previously described by Kim et al. [27].

### 2.5. Inflammatory Status, Humoral Immune Status, and Oxidative Stress Parameters

Frozen 1 g jejunal mucosa was taken with 2 mL phosphate-buffered saline solution (PBS) into 5 mL polypropylene tubes. Mucosa samples were ground for 30 s on ice and transferred to new 2 mL tubes for centrifugation of 15 min at 3000× *g*. The supernatant was collected into eight sets of 0.5 mL polypropylene tubes and stored at −80 °C. Sample preparation for analysis was followed as previously described by Holanda and Kim [28].

The concentrations of total protein, tumor necrosis factor-alpha (TNF-α), interleukin 8 (IL-8), malondialdehyde (MDA), protein carbonyl (PC), immunoglobulin A (IgA), and immunoglobulin G (IgG) were measured by the colorimetric method using commercially available kits according to instructions of the manufacturers. For each assay, the absorbance was read using an ELISA plate reader (Synergy HT, BioTek Instruments, Winooski, VT, USA) and software (Gen5 Data Analysis Software, BioTek Instruments). Mucosa samples were diluted (1:60) for the measurement of total protein in the working range 20–2000 μg/mL using Pierce BCA Protein Assay Kit (#23225, Thermo Fisher Scientific). The absorbance was measured at 562 nm and the concentration of protein was calculated based on the standard curve created from the concentration and absorbance of the respective standard. The total protein concentration was further used to normalize the concentration of the others parameters.

The TNF-α was measured using the kit Porcine TNF-α Immunoassay Kit (#PTA00, R&D Systems; Minneapolis, MN, USA). The working range of standards was 0 to 1500 pg/mL, and the absorbance was read at 450 nm and 550 nm. The IL-8 was measured following Porcine IL-8/CXCL8 Immunoassay Kit (#P8000, R&D Systems). For this analysis, mucosa samples were diluted (1:10) in a working range of 0–4000 pg/mL and the absorbance was read at 450 nm and 550 nm. The concentrations of TNF-α and IL-8 were calculated based on the standard curve created from concentration and absorbance of the respective standard and described as pg/mg of protein, following Holanda and Kim [28].

Malondialdehyde was measured following OxiSelect TBARS MDA Quantitation Assay Kit (#STA-330, Cell Biolabs, Inc.; San Diego, CA, USA). The concentration range of MDA standards was 0–125 μM. The absorbance was measured at 540 nm. The concentration of MDA was calculated based on the standard curve created from the concentration and absorbance of the respective standard and described as μmol/mg of protein, as described by Jang and Kim [29].

Protein carbonyl was measured following OxiSelect Protein Carbonyl ELISA Kit (#STA-310, Cell Biolabs, Inc). All samples were diluted to 10 μg/mL of PBS solution. The working range of standards was 0.375–7.500 nmol/mg protein. The absorbance was measured at 540 nm. The concentration of protein carbonyl was calculated based on the standard curve created from concentration and absorbance of the respective standard and described as nmol/mg of protein, following Duarte et al. [2].

The concentrations of IgG and IgA were analyzed following Chen et al. [30]. The mucosa samples were diluted with PBS to 1:1000, to analyze IgG using the kit ELISA Pig IgG (E101-104, Bethyl Laboratories, Inc, Montgomery, TX). The standard was used in a working range for 0–500 ng/mL. The absorbance was measured at 450 nm and the concentration of IgG in the mucosa was expressed as ng/mg of protein. The mucosal samples were diluted to 1:600 with PBS to analyze IgA using the kit ELISA Pig IgA (E101-102, Bethyl Laboratories, Inc). The standard was used in a working range of 0–1000 ng/mL. The absorbance was measured at 450 nm and the concentration of IgA in the mucosa was expressed as µg/mg of protein.

### 2.6. Intestinal Morphology

After being fixed in 10% formalin for 48 h, the jejunal tissue was rinsed with deionized water and then transferred to a 70% ethanol solution. Two sections of the jejunum were cut transversely and placed in cassettes into a container with 70% ethanol solution. The samples were sent to the North Carolina State University Histology Laboratory (Raleigh, NC). Then, the samples were dehydrated, embedded in paraffin, cut cross-section to 5 µm thick, and mounted on polylysine-coated slides. Slides were stained using a Ki-67 immunohistochemistry assay to detect Ki-67 positive cells as previously described by Kim et al. [27]. Villus height, villus width, and crypt depth were measured using a microscope (Olympus CX31, Lumenera Corporation, Ottawa, Canada) attached to a camera (Infinity 2-2 digital CCD, Lumenera Corporation). Lengths of 10 well-oriented intact villi and their associated crypts were measured in each slide. The villi length was measured from the top of the villi to the villi-crypt junction, the villi width measured in the middle of the villi, and the crypt depth was measured from the villi-crypt junction to the bottom of the crypt. Then, the villus height to crypt depth ratio (VH:CD) was calculated. Images of 10 intact crypts from each slide were cropped and the Image JS software was used for calculating the ratio of Ki-67 positive cells to total cells in the crypt (%). The averages of the 10 measurements per pig were calculated and reported as one number per pig. All analyses of the intestinal morphology were executed by the same person as previously described by Sun et al. [31]. The villus surface area was calculated using the following formula: villus surface area = 2π × (villus width) × (villus height). The means were expressed as ×100 µm^2^ as previously described by Xu et al. [32].

### 2.7. Apparent Ileal Digestibility

The frozen ileal digesta samples were dried by a freeze dryer. Dried digesta and feed samples were ground to fine powder form and stored into plastic containers for further analysis. Titanium dioxide concentration in the feed and digesta was measured as previously described by Myers et al. [33]. The working range of the standards was 0–10 mg of titanium dioxide. Samples were weighed around 0.5 g onto a tarred weighing paper and then placed into 75 mL digestion tubes. One Kjeltab tablet (Thermo Fisher Scientific) and five pieces of selenized boiling granules were added to each digestion tube to prevent explosive vaporization. After adding 10 mL of concentrated H_2_SO_4_ (sulfuric acid), all digestion tubes were vortexed immediately. Then, the tubes were heated for 2.5 h at 420 °C under a fume hood. When tubes got cool after 30 min at room temperature, 2 mL of 30% H_2_O_2_ (hydrogen peroxide) was added to each tube four times and was vortexed until a yellow to orange color appeared. Deionized water was added until reaching the volumetric mark (75 mL) and then the tubes were covered and gently mixed. After that, 200 µL from each tube was pipetted to a 96-well plate, which was read immediately at 410 nm. Titanium dioxide values were calculated based on the standard curve created from the concentration and absorbance of the respective standards.

The feed and digesta samples were weighed around 0.5 g to analyze the nitrogen content using TruSpec N Nitrogen Determinator (LECO CN-2000, LECO Corp., St. Joseph, MI, USA) to later obtain the CP (6.25 × N). Feed and digesta samples were weighed around 1 g and then compressed into pellet form to measure gross energy via bomb calorimeter (Parr 6200, Parr Instrument Company; Moline, IL, USA). Apparent ileal digestibility of DM, CP, and GE was calculated using the following equation previously described by Chen et al. [34]:

AID (%) = {1 − [(TiO_2feed_/TiO_2digesta_) × (Nutrient_digesta_/Nutrient_feed_)]} × 100 (1)

where TiO_2feed_ represents the titanium dioxide concentration in the feed, TiO_2digesta_ is the titanium dioxide concentration in the ileal digesta, Nutrient_feed_ represents the nutrient concentration or GE in the feed, and Nutrient_digesta_ is the nutrient concentration or GE in the ileal digesta.

### 2.8. Statistical Analysis

Data were analyzed based on a randomized block design by the SAS 9.4 software (SAS Inc., Cary, NC, USA). Dietary treatments were defined as fixed effects; random effects were blocks. The experimental unit was the pig, individually housed and fed. The analyses of the data of growth performance, digestibility, morphology, and immune and oxidative markers were performed using the MIXED procedure. Linear and quadratic effects of increasing supplementation of functional oils were tested by polynomial contrasts (*n* = 40). A pre-planned contrast was established to compare the inclusion of functional oils and the negative control diets (0 vs. others). The IML procedure was used to generate the coefficients for orthogonal polynomials. The RSREG procedure was used to predict the critical value and the stationary point when a quadratic effect was found. The fecal score data were analyzed using the MIXED procedure using the repeated measures statement as previously described in Duarte et al. [25]. Treatment, time, and their interaction were used as fixed effects. Initial BW and sex were considered random effects. When an interaction between treatment and time resulted, the comparisons among treatments means at different time points were performed using the slice command. Statistical differences were considered significant with *p* < 0.05 and tendency with 0.05 ≤ *p* < 0.10. The ls means statement was used to calculate the least squared mean values of normally distributed data. The microbiome data were tested for normal distribution with the UNIVARIATE (Shapiro-Wilk test), and the non-normally distributed data were analyzed using the GLIMMIX procedure through Poisson distributions according to Zhang et al. [35]. Dietary treatments were defined as fixed effects and the initial BW blocks within each sex were considered as random effects. The polynomial contrasts (linear, quadratic, and 0 vs others) effects were tested with multiple testing corrections applied according to Benjamini-Hochberg’s false discovery rate (q-value). A *p* value of <0.05 with a q value of <0.05 was considered significant. Results on Poisson distributed data are presented as back-transformed estimates with a 95% confidence interval.

## 3. Results

### 3.1. Growth Performance and Fecal Score

Increasing supplementation of functional oils did not affect BW, ADG, and ADFI of nursery pigs during the experimental period (Table 2). However, increasing supplementation of functional oils had a quadratic effect (*p* < 0.05) on G:F (minimum: 0.62 at 1.00 g/kg feed) during phase 1. Supplementation of functional oils (0.50 to 1.50 g/kg feed) reduced (*p* < 0.05) G:F of phase 1 compared with no supplementation.

Increasing supplementation of functional oils did not affect the fecal score of the pigs, whereas there was an effect of time on the treatment containing 1.50 g/kg feed functional oils (*p* < 0.05); the fecal score on the periods (day 1–7 and day 8–13) were greater than period (day 28–34). However, there was no interaction between treatments and time (Table 3).

### 3.2. Microbiota

At the phylum level (Table 4), increasing supplementation of functional oils increased (*p* < 0.05) the relative abundance of Firmicutes and Bacteroidetes and tended to decrease (*p* = 0.054) the Firmicutes/Bacteroidetes ratio. Whereas, increasing supplementation of functional oils reduced (*p* < 0.05) the relative abundance of Proteobacteria and increased (*p* < 0.05) the relative abundance of Firmicutes.

At the family level (Table 5), increasing supplementation of functional oils reduced (*p* < 0.05) the relative abundance of Helicobacteraceae and increased (*p* < 0.05) the relative abundance of Prevotellaceae, Burkholderiaceae, and Pseudomonadaceae. Whereas, increasing supplementation of functional oils had a quadratic effect (*p* < 0.05) on the relative abundance of Comamonadaceae (minimum: 2.94% at 0.61 g/kg feed) and Enterobacteriaceae (minimum: 0.82% at 0.62 g/kg feed) and tended to have a quadratic effect on the relative abundance of Burkholderiaceae (*p* = 0.083) (minimum: 5.93% at 0.61 g/kg feed) and Pseudomonadaceae (*p* = 0.052) (minimum: 3.22% at 1.43 g/kg feed). Moreover, increasing supplementation of functional oils (0.50 to 1.50 g/kg feed) reduced (*p* < 0.05) the relative abundance of Helicobacteraceae and Enterobacteriaceae and tended to increase (*p* = 0.052) the relative abundance of Pseudomonadaceae compared with no supplementation.

At the genus level (Table 6), increasing supplementation of functional oils reduced (*p* < 0.05) the relative abundance of *Helicobacter* and increased (*p* < 0.05) the relative abundance of *Pelomonas* and *Pseudomonas*. Whereas, increasing supplementation of functional oils had a quadratic effect (*p* < 0.05) on the relative abundance of *Helicobacter* (minimum: 34.73% at 0.80 g/kg feed), *Campylobacter* (maximum: 2.03% at 0.12 g/kg feed), *Pseudomonas* (maximum: 5.31% at 0.63 g/kg feed), and *Corynebacterium* (maximum: 2.27% at 0.58 g/kg feed). Moreover, increasing supplementation of functional oils (0.50 to 1.50 g/kg feed) decreased (*p* < 0.05) the relative abundance of *Helicobacter* and *Campylobacter*; increased (*p* < 0.05) the relative abundance of *Lactobacillus*, *Pseudomonas*, and “others” (representing the combined OTU with the relative abundance < 1%); and tended to reduce (*p* = 0.076) the relative abundance of *Corynebacterium* compared with no supplementation.

At the species level (Table 7), increasing supplementation of functional oils reduced (*p* < 0.05) the relative abundance of *Helicobacter rappini* and *Helicobacter mastomyrinus,* and increased (*p* < 0.05) the relative abundance of *Prevotella copri*, *Lactobacillus kitasatonis*, *Curpiavidus necator,* and *Acinetobacter radioresistens*. Increasing supplementation of functional oils tended to reduce (*p* = 0.075) the relative abundance of *Pelomonas puraquae*. Whereas, increasing supplementation of functional oils had a quadratic effect (*p* < 0.05) on the relative abundance of *Helicobacter mastomyrinus* (maximum: 15.71% at 1.05 g/kg feed), *Pelomonas puraquae* (minimum: 4.59% at 0.83 g/kg feed), *Ralstonia insidiosa* (maximum: 3.78% at 0.42 g/kg feed) and “others” (maximum: 34.10% at 1.35 g/kg feed). Moreover, increasing supplementation of functional oils (0.50 to 1.50 g/kg feed) reduced (*p* < 0.05) the relative abundance of *Helicobacter rappini*, *Helicobacter mastomyrinus,* and *Helicobacter* sp.; and increased (*p* < 0.05) the relative abundance of *Prevotella copri* and “others” compared with no supplementation. Also, it tended to reduce (*p* = 0.066) the relative abundance of *Prevotella ruminicola*.

In the same way, increasing supplementation of functional oils increased (*p* < 0.05) the alpha diversity of the mucosa-associated microbiota at the family level estimated with the Chao1 index (Table 8) and tended to increase (*p* = 0.053) the alpha diversity of the mucosa-associated microbiota at the genus level estimated with the Chao1 index.

### 3.3. Immune Status and Oxidative Stress

Increasing supplementation of functional oils did not affect the immune and oxidative stress and status parameters. However, the supplementation of functional oils (0.50 to 1.50 g/kg feed) tended to decrease (*p* = 0.064) the concentration of protein carbonyl compared with no supplementation (Table 9).

### 3.4. Intestinal Morphology

Increasing supplementation of functional oils (0.50 to 1.50 g/kg feed) tended to increase (*p* = 0.098) the villus height and crypt depth (*p* = 0.070) compared with no supplementation (Table 10). Whereas, increasing supplementation of functional oils did not affect other analyzed parameters.

### 3.5. Apparent Ileal Digestibility

Increasing supplementation of functional oils did not affect the AID of DM, CP, and GE (Table 11).

## 4. Discussion

In this study, increasing supplementation of functional oils did not affect the fecal score, whereas the fecal score of pigs on treatment containing 1.50 g/kg feed was better at the last week of the experiment in comparison with phase 1, indicating that beneficial effects on reducing fecal score were obtained about 4 weeks after feeding diets with the blend of functional oils at the level of 1.50 g/kg.

Although, some laxative effects have been reported to be orally administered [21], the cashew nutshell liquid increases the antimicrobial property of castor oil, allowing a reduction on the castor oil levels without losing the benefits associated with the anti-inflammatory and antimicrobial activities [22,36], therefore, suppressing the laxative effects [19,20]. Additionally, supplementation of functional oils resulted in improving feed efficiency of nursery pigs during phase 1, without affecting the growth performance in the overall experimental period. However, in previous studies utilizing the same blend of castor oil and cashew nutshell liquid, Murakami et al. [19] and Bess et al. [20] observed an increase in ADG and improved feed efficiency of broiler chickens fed a diet supplemented at 1.50 g/kg feed. Likewise, Torrent et al. [17] tested the effect of functional oils supplemented at 1.50 g/kg feed for broiler chickens under heat stress and also observed an increase in ADG by the end of the trial. In the same way, Moraes et al. [37] observed that supplementation of functional oils can provide positive effects on the growth performance of broiler chickens. The authors correlated the positive effects of the functional oils, composed by a blend of castor oil, and cashew nutshell liquid, to their antimicrobial, anti-inflammatory, and antioxidant properties.

During the nursery phase, continual changes of the intestinal microbiome take place in response to various bacteria that come in contact with the host, disease, stressor levels, dietary changes [38], and feed additive supplementation [27]. In the present study, the supplementation of functional oils resulted in an increase in the relative abundance of *Lactobacillus* and *Pseudomonas*, both of which belong to the Firmicutes and Bacteroidetes phyla, respectively, and a decrease in the relative abundance of *Helicobacter* and *Campylobacter*, belonging to Proteobacteria. The changes in the microbiota population observed in the present study may be due to cardol and anacardic acid present in cashew nutshell liquid that acts as monovalent ionophores, causing damage to the cell membrane of gram-negative bacteria [39,40] and consequently reducing their abundance. In addition, the anacardic acid would act to enhance the neutrophil antibacterial function by promoting the production of neutrophil extracellular traps (NETs) [41], which are DNA-based structures that play a key role in pathogen clearance by neutrophils [42]. Additionally, the ratio between Firmicutes and Bacteroidetes was reduced with increasing supplementation of functional oils in the current study, which can be beneficial to the pigs [43]. Furthermore, increasing the supplementation of functional oils improved the alpha diversity of jejunal mucosa-associated microbiota at the family and genus levels, which is associated with beneficial effects on the intestinal health [44,45]. It is well known that the balance of microbiota is related to the greater diversity of species [26]. Greater microbiota diversity have been related to downregulated inflammatory response in the intestinal mucosa of nursery pigs [46,47], although this was not observed in the current study.

According to [20], the benefits of increasing supplementation of functional oils relative to the growth performance may be related to the anti-inflammatory and antioxidant properties of the blend. In the present study, the oxidative stress status was affected by a reduction in the PC concentration, without affecting the MDA and the anti-inflammatory parameters. The imbalance between the antioxidants and free radicals in the body characterizes oxidative stress [48]. In response, the organism produces different biomarkers, such as cytokines and immunoglobulins, to reduce and identify cell damage caused by the free radicals [29,30,48]. Reactive oxygen species (ROS) are formed as a natural byproduct from the metabolism of oxygen and play important roles in cell signaling and homeostasis. However, in high or chronic stress situations, an overproduction of ROS can occur and lead to increased oxidation and damage to the cell as well as cause an imbalance in the activation of the immune response [49,50,51]. High levels of ROS can degrade polyunsaturated lipids and protein groups, forming MDA and PC, both being toxic to the organism [51,52]. The tendency to reduce the concentration of PC with increasing supplementation of functional oils may be related to the roles of the compounds in functional oils that possess anti-inflammatory [18] and antioxidant properties [23] that can alleviate the effects of oxidative stress. The antioxidant action of the functional oils is due to the properties of the compounds within cashew nutshell liquid, which increases their rate of reaction with peroxyl radicals, a toxic compound to the cell, thus increasing the autoxidation of these toxic compounds [23]. Ricinoleic acid, the main compound in castor oil, has been shown to modulate neurogenic inflammation through a capsaicin-like action [18]. The capsaicin and capsaicin-like compounds stimulate the release of sensory neuropeptides from the peripheral endings of primary afferent neurons by rendering them insensitive to further stimulation and thereby presenting inflammatory and anti-inflammatory effects [18,53,54]. At this later stage of capsaicin and capsaicin-like action, an anti-inflammatory effect is caused in response to endogenous or exogenous neurogenic inflammation [18,54].

The oxidative stress products can affect proteins and lipids within the cell wall, leading to cell destruction and, consequently, changes in jejunal morphology [2,55,56]. This study showed that increasing supplementation of functional oils tended to increase the villus height and crypt depth in the mid-jejunum, without affecting other morphology parameters. Ferket et al. [16] tested the same source of functional oils and did observe an increase in the villus height and crypt depth of turkey poults. Therefore, the tendency of the reduction of the oxidative stress status found in the current study due to increasing supplementation of functional oils can partially explain the improvement in jejunal morphology.

The benefits related to the modulation of the intestinal mucosa-associated microbiota may have played a role in the positive effects observed on the oxidative stress status that in turn led to an enhancement of the jejunal morphology. Even without positively affecting the growth performance, increasing supplementation of functional oils positively modulated the intestinal mucosa-associated microbiota by decreasing the relative abundance of some potential pathogenic bacteria and increasing the relative abundance of beneficial bacteria. Beneficial modulation of the intestinal mucosa-associated microbiota can be essential for overall nursery pig performance by improving oxidative stress and immune status as well as intestinal morphology measurements [26,27].

## 5. Conclusions

In conclusion, the supplementation of functional oils enhanced intestinal health of nursery pigs by increasing beneficial and reducing harmful bacteria in the jejunal mucosa, and by potentially reducing jejunal oxidative stress and maintaining villus height, without affecting overall growth performance. Supplementation of functional oils at 0.75–1.50 g/kg feed was most beneficial to the structure of the jejunal mucosa-associated microbiota and intestinal integrity of nursery pigs.

## Figures and Tables

**Table 1:** Composition of basal diets (as-fed basis).

Item	Phase 1	Phase 2
Ingredient, %		
Corn, yellow	46.06	49.97
Soybean meal, 48% CP	19.00	21.00
Corn, DDGS ^1^	10.50	15.00
Whey permeate	12.50	6.25
Blood plasma	3.00	1.00
Poultry meal	4.10	2.00
Poultry fat	1.80	1.50
L-Lys HCl	0.56	0.51
DL-Met	0.17	0.12
L-Thr	0.15	0.12
L-Trp	0.01	0.01
Salt	0.23	0.22
Vitamin premix ^2^	0.03	0.03
Trace mineral premix ^3^	0.15	0.15
Dicalcium phosphate	0.62	0.55
Limestone	0.96	1.02
Titanium dioxide	0.00	0.40
Calculated composition		
Dry matter, %	90.18	89.38
ME, kcal/kg	3404	3370
Crude protein, %	21.82	21.14
SID ^4^ Lys, %	1.35	1.23
SID Cys + Met, %	0.74	0.68
SID Trp, %	0.22	0.20
SID Thr, %	0.79	0.73
Ca, %	0.80	0.70
STTD ^5^ P, %	0.40	0.33
Total P, %	0.63	0.65
Analyzed composition		
Dry matter, %	88.97	88.68
Crude protein, %	22.30	22.11
Ca, %	0.96	0.75
Total P, %	0.72	0.68

^1^ DDGS: distillers dried grains with solubles. ^2^ The vitamin premix provided per kilogram of complete diet: 6614 IU of vitamin A as vitamin A acetate, 992 IU of vitamin D_3_, 19.8 IU of vitamin E, 2.64 mg of vitamin K as menadione sodium bisulfate, 0.03 mg of vitamin B_12_, 4.63 mg of riboflavin, 18.52 mg of D-pantothenic acid as calcium pantothenate, 24.96 mg of niacin, and 0.07 mg of biotin. ^3^ The trace mineral premix provided per kilogram of complete diet: 39.6 mg of Mn as manganous oxide; 165 mg of Fe as ferrous sulfate; 165 mg of Zn as Zinc sulfate; 15.15 mg of Cu as copper sulfate; 0.30 mg of I as ethylenediamine dihydroiodide; and 0.30 mg of Se as sodium selenite. ^4^ Functional oils composed of a mixture of castor oil and cashew nutshell liquid containing: oleic acid: 4.51%; palmitic acid: 1.30%; stearic acid: 1.38%; 8,11-Octadecadienoic acid methyl ester: 4.39%; Octadeca-9,12,15 trienoic acid: 0.54%; methyl ricinoleate: 87.37%; and elaidic acid: 0.51% (Oligo Basics, Cary, CA, USA) or corn. ^4^ SID = standardized ileal digestible. ^5^ STTD = standardized total tract digestible.

**Table 2:** Growth performance of nursery pigs fed diets with increasing supplementation of functional oils.

Item	Functional Oils, g/kg Feed ^1^						*p*-Value		
	0.00	0.50	0.75	1.00	1.50	SEM	Linear	Quadratic	0 vs. Others
BW, kg									
Initial	7.0	7.0	7.0	7.0	7.0	0.5	0.922	0.988	0.913
d 13	9.3	9.0	9.3	9.3	9.5	0.8	0.432	0.554	0.899
d 34	22.5	22.0	23.1	22.0	23.6	1.6	0.205	0.570	0.787
ADG, g/d									
Phase 1	173	149	174	160	194	30	0.261	0.491	0.859
Phase 2	628	617	658	617	669	40	0.223	0.693	0.659
Overall	454	441	473	442	488	35	0.166	0.535	0.753
ADFI, g/d									
Phase 1	205	237	258	253	247	43	0.392	0.284	0.161
Phase 2	926	910	997	953	1031	75	0.109	0.938	0.426
Overall	650	652	715	685	731	61	0.114	0.720	0.291
G:F									
Phase 1	0.85	0.63	0.66	0.65	0.79	0.05	0.758	0.004	0.003
Phase 2	0.68	0.69	0.66	0.65	0.66	0.02	0.216	0.423	0.450
Overall	0.70	0.68	0.67	0.65	0.67	0.02	0.319	0.127	0.141

^1^ Five supplemental levels of functional oils (*n* = 40 total, *n* = 8 per supplemental level); Results are showed as least squared means.

**Table 3:** Fecal score of nursery pigs fed diets with increasing supplementation of functional oils.

Item	Functional Oils, g/kg Feed ^1^						*p*-Value	
	0.00	0.50	0.75	1.00	1.50	SEM	Trt ^2^	Trt × Time
d 0 to 7	3.16	3.33	3.28	3.25	3.37 ^a^	0.11	0.661	
d 7 to 13	3.20	3.12	3.24	3.37	3.41 ^a^	0.10	0.262	
d 13 to 20	3.07	3.16	3.21	3.35	3.23 ^a,b^	0.09	0.240	
d 20 to 27	3.10	3.07	3.19	3.19	3.21 ^a,b^	0.07	0.347	
d 27 to 34	3.20	3.12	3.10	3.21	3.07 ^b^	0.08	0.569	
*p* value, Time	0.723	0.296	0.600	0.492	0.049			0.726

^1^ Five supplemental levels of functional oils (*n* = 40 total, *n* = 8 per supplemental level). Results are showed as least squared means. ^2^ Trt = treatments. ^a,b^ Within a column with different letters differ (*p* < 0.05).

**Table 4:** Relative abundance of jejunal mucosa-associated microbiota at the phylum level in nursery pigs fed diets with increasing supplementation of functional oils.

Item	Functional Oils, g/kg Feed ^1^						*p*-Value		
	0.00	0.50	0.75	1.00	1.50	SEM	Linear	Quadratic	0 vs. Others
Proteobacteria	73.50	59.22	52.26	68.31	60.16	6.12	0.313	0.205	0.009
Firmicutes	11.97	20.50	22.12	17.17	23.32	4.24	0.013	0.258	0.009
Bacteroidetes	8.16	8.57	11.99	11.83	14.37	2.14	0.012	0.304	0.050
Actinobacteria	2.96	7.52	3.55	2.72	3.31	1.43	0.272	0.657	0.305
Others ^2^	0.70	0.45	4.91	0.27	0.66	0.83	0.658	0.635	0.843
F:B ^3^	2.08	4.04	2.30	1.42	1.10	0.56	0.054	0.979	0.870

^1^ Five supplemental levels of functional oils (*n* = 40 total, *n* = 8 per supplemental level). Results showed as means presented as back-transformed estimates with a 95% confidence interval. ^2^ Representing the combined total of all <1% abundance. ^3^ Firmicutes to Bacteroidetes ratio.

**Table 5:** Relative abundance of jejunal mucosa-associated microbiota at the family level in nursery pigs fed diets with increasing supplementation of functional oils.

Item	Functional Oils, g/kg Feed ^1^						*p*-Value		
	0.00	0.50	0.75	1.00	1.50	SEM	Linear	Quadratic	0 vs. Others
Helicobacteraceae	46.75	40.49	26.46	48.43	20.95	10.76	0.002	0.139	0.002
Lactobacillaceae	7.09	10.81	15.60	6.58	12.42	1.58	0.314	0.966	0.138
Prevotellaceae	7.87	7.56	11.00	11.15	13.10	2.32	0.009	0.485	0.314
Burkholderiaceae	6.32	5.28	7.05	4.24	10.82	1.05	0.043	0.083	0.957
Comamonadaceae	4.36	2.50	3.89	1.73	5.81	0.72	0.314	0.045	0.439
Veillonellaceae	2.44	3.23	2.16	2.17	2.90	1.02	0.930	0.593	0.931
Enterobacteriaceae	4.05	0.93	1.4	0.99	2.93	0.97	0.639	0.007	0.004
Microbacteriaceae	1.86	1.12	1.62	0.91	2.31	0.47	0.593	0.314	0.639
Streptococcaceae	0.97	1.59	1.15	0.76	2.49	0.66	0.317	0.423	0.655
Lachnospiraceae	0.74	1.08	1.2	1.44	1.58	0.45	0.408	0.655	0.593
Clostridiaceae	0.53	1.62	0.57	0.68	1.36	0.49	0.609	0.653	0.639
Campylobacteraceae	0.34	1.13	1.51	0.10	0.53	0.59	0.593	0.655	0.639
Pseudomonadaceae	0.07	0.33	0.75	0.76	0.98	0.82	0.005	0.052	0.052
Moraxellaceae	0.10	0.24	0.51	0.10	1.17	0.40	0.083	0.655	0.510
Others ^2^	5.07	9.96	6.21	4.92	7.22	3.23	0.957	0.553	0.382

^1^ Five supplemental levels of functional oils (*n* = 40 total, *n* = 8 per supplemental level). Results showed as means presented as back-transformed estimates with a 95% confidence interval. ^2^ Representing the combined total of all <1% abundance.

**Table 6:** Relative abundance of jejunal mucosa-associated microbiota at the genus level in nursery pigs fed diets with increasing supplementation of functional oils.

Item	Functional Oils, g/kg Feed ^1^						*p*-Value		
	0.00	0.50	0.75	1.00	1.50	SEM	Linear	Quadratic	0 vs. Others
*Helicobacter*	42.44	28.84	51.14	23.33	50.42	10.89	0.016	0.002	0.041
*Lactobacillus*	11.36	16.47	9.63	13.39	9.97	2.09	0.990	0.365	0.013
*Prevotella*	7.78	11.39	10.96	13.38	6.95	2.14	0.958	0.728	0.813
*Ralstonia*	4.64	5.85	3.65	9.48	6.44	0.94	0.378	0.292	0.114
*Pelomonas*	2.72	4.26	1.88	6.56	4.75	0.69	0.003	0.114	0.292
*Microbacterium*	1.26	1.76	1.01	2.68	2.13	0.46	0.782	0.435	0.252
*Cupriavidus*	0.69	1.33	0.82	2.54	1.24	0.41	0.252	0.285	0.369
*Campylobacter*	1.28	1.57	0.12	0.76	0.23	0.66	0.358	0.020	0.035
*Pseudomonas*	0.32	0.70	0.75	1.00	0.85	1.09	0.016	0.015	0.007
*Corynebacterium*	1.61	0.07	0.06	0.02	0.01	0.94	0.285	0.002	0.076
*Acinetobacter*	0.10	0.38	0.07	0.99	0.13	0.38	0.127	0.292	0.817
Others ^2^	8.29	6.45	6.71	8.67	5.16	2.96	0.159	0.284	0.217

^1^ Five supplemental levels of functional oils (*n* = 40 total, *n* = 8 per supplemental level). Results showed as means presented as back-transformed estimates with a 95% confidence interval. ^2^ Representing the combined total of all <1% abundance.

**Table 7:** Relative abundance of jejunal mucosa-associated microbiota at the species level in nursery pigs fed diets with increasing supplementation of functional oils.

Item	Functional Oils, g/kg Feed ^1^						*p*-Value		
	0.00	0.50	0.75	1.00	1.50	SEM	Linear	Quadratic	0 vs. Others
*Helicobacter rappini*	28.60	26.26	17.81	18.43	13.78	2.76	0.001	0.146	0.001
*Helicobacter mastomyrinus*	15.98	8.94	17.09	14.40	6.62	2.64	0.001	0.002	0.004
*Prevotella copri*	7.57	10.89	9.57	13.98	16.20	1.73	0.001	0.406	0.005
*Pelomonas puraquae*	5.45	3.62	4.74	2.66	7.92	0.77	0.075	0.009	0.328
*Lactobacillus mucosae*	2.68	4.94	4.83	4.22	3.02	1.04	0.644	0.102	0.146
*Ralstonia insidiosa*	3.96	3.00	3.45	1.59	4.32	0.84	0.829	0.040	0.232
*Prevotella sp.*	3.04	1.13	2.31	3.07	2.67	0.89	0.328	0.828	0.232
*Microbacterium ginsengisoli*	2.60	1.85	2.10	1.44	3.69	0.53	0.228	0.102	0.535
*Prevotella ruminicola*	2.50	0.51	1.50	2.52	1.88	0.97	0.228	0.800	0.066
*Pelomonas aquatica*	1.61	1.40	1.57	0.98	2.82	0.45	0.209	0.210	0.948
*Lactobacillus kitasatonis*	0.73	1.25	1.64	0.54	4.21	0.58	0.002	0.211	0.218
*Propionibacterium acnes*	0.63	2.19	1.19	0.69	0.48	0.50	0.211	0.545	0.454
*Streptococcus alactolyticus*	1.02	1.23	0.59	0.60	1.66	0.57	0.406	0.115	0.825
*Cupriavidus necator*	0.72	0.36	0.69	0.93	2.12	0.48	0.005	0.584	0.805
*Helicobacter sp.*	1.98	0.43	0.39	1.27	0.06	0.52	0.102	0.377	0.001
*Corynebacterium glutamicum*	0.01	0.92	0.03	0.01	0.01	0.26	0.524	0.825	0.406
*Acinetobacter radioresistens*	0.05	0.04	0.14	0.03	0.68	0.30	0.001	0.200	0.345
Others ^2^	13.00	19.90	16.32	25.92	17.44	3.56	0.218	0.005	0.006

^1^ Five supplemental levels of functional oils (*n* = 40 total, *n* = 8 per supplemental level). Results showed as means presented as back-transformed estimates with a 95% confidence interval. ^2^ Representing the combined total of all <1% abundance.

**Table 8:** Alpha diversity of jejunal mucosa-associated microbiota at the family level in nursery pigs fed diets with increasing supplementation of functional oils.

Item	Functional Oils, g/kg Feed ^1^						*p*-Value		
	0.00	0.50	0.75	1.00	1.50	SEM	Linear	Quadratic	0 vs. Others ^2^
Family									
Chao1	35.99	47.70	44.05	48.87	58.99	26.85	0.044	0.966	0.092
Shannon	2.36	2.41	2.83	2.21	2.88	0.92	0.249	0.672	0.474
Simpson	0.60	0.57	0.70	0.54	0.72	0.18	0.174	0.486	0.676
Genus									
Chao1	35.40	47.73	40.45	49.14	56.86	26.53	0.053	0.983	0.103
Shannon	2.10	2.23	2.60	1.98	2.64	0.89	0.276	0.712	0.414
Simpson	0.54	0.53	0.67	0.49	0.67	0.19	0.218	0.677	0.506

^1^ Five supplemental levels of functional oils (*n* = 40 total, *n* = 8 per supplemental level); results are shown as least squared means. ^2^ Representing the combined total of all <1% abundance.

**Table 9:** Immune status and oxidative stress of nursery pigs fed diets with increasing supplementation of functional oils.

Item	Functional Oils, g/kg Feed ^1^						*p*-Value		
	0.00	0.50	0.75	1.00	1.50	SEM	Linear	Quadratric	0 vs. FO
TNF-α ^2^, pg/mg of protein	0.47	0.54	0.43	0.37	0.64	0.09	0.323	0.130	0.810
IL-8 ^3^, pg/mg of protein	650	625	530	558	771	117	0.388	0.200	0.825
MDA ^4^, µmol/mg of protein	0.57	0.70	0.57	0.61	0.57	0.19	0.734	0.925	0.765
PC ^5^, nmol/mg of protein	3.11	2.42	2.83	2.45	2.55	0.37	0.304	0.421	0.064
IgA ^6^, µg/mg of protein	3.41	4.17	4.32	3.69	3.75	0.70	0.941	0.645	0.497
IgG ^7^, µg/mg of protein	2.42	1.71	1.65	2.43	2.02	0.53	0.980	0.893	0.432

^1^ Five supplemental levels of functional oils (*n* = 40 total, *n* = 8 per supplemental level); results are shown as least squared means. ^2^ Tumor necrosis factor-alpha; ^3^ Interleukin 8; ^4^ Malondialdehyde; ^5^ Protein carbonyl; ^6^ Immunoglobulin A; ^7^ Immunoglobulin G.

**Table 10:** Jejunal morphology in nursery pigs fed diets with increasing supplementation of functional oils.

Item	Functional Oils, g/kg Feed ^1^						*p*-Value		
	0.00	0.50	0.75	1.00	1.50	SEM	Linear	Quadratic	0 vs. Others
Villus height, μm	401	471	451	416	453	25	0.578	0.977	0.098
Villus width, μm	97	90	91	94	93	3	0.921	0.568	0.196
Crypt depth, μm	86	90	99	97	96	7	0.166	0.105	0.070
Villus area, μm^2^ × 10^−2^	385	420	407	390	417	20	0.574	0.800	0.303
VH:CD ratio ^2^	4.7	5.2	4.6	4.4	4.7	0.3	0.435	0.282	0.751
Ki-67 positive ^3^, %	36.7	36.7	39.3	37.6	37.3	2.3	0.748	0.289	0.463

^1^ Five supplemental levels of functional oils (*n* = 40 total, *n* = 8 per supplemental level); Results are shown as least squared means. ^2^ Villus height to crypt depth ratio; ^3^ Ratio of Ki-67 positive cells to total cells in the crypt.

**Table 11:** Apparent ileal digestibility of dry matter, crude protein, and gross energy in diets of nursery pigs with increasing supplementation of functional oils.

Item	Functional Oils, g/kg Feed ^1^						*p*-Value		
	0.00	0.50	0.75	1.00	1.50	SEM	Linear	Quadratic	0 vs. Others ^2^
Dry matter, %	61.05	62.87	60.71	63.32	63.96	3.24	0.500	0.950	0.562
Crude protein, %	68.29	68.46	71.72	80.06	67.88	6.10	0.856	0.145	0.534
Gross energy, %	65.56	64.81	66.87	68.17	65.59	3.16	0.867	0.497	0.812

^1^ Five supplemental levels of functional oils (*n* = 40 total, *n* = 8 per supplemental level); Results are showed as least squared means. ^2^ Representing the combined total of all <1% abundance.

## Data Availability

The data presented in this study are available upon request from the corresponding author.
